# To Bud or Not to Bud: A Perspective on Molecular Simulations of Lipid Droplet Budding

**DOI:** 10.3389/fmolb.2019.00124

**Published:** 2019-11-13

**Authors:** Valeria Zoni, Vincent Nieto, Laura J. Endter, Herre J. Risselada, Luca Monticelli, Stefano Vanni

**Affiliations:** ^1^Department of Biology, University of Fribourg, Fribourg, Switzerland; ^2^Molecular Microbiology and Structural Biochemistry, UMR 5086 CNRS, Universitè de Lyon, Lyon, France; ^3^Department of Theoretical Physics, Georg-August University Göttingen, Göttingen, Germany; ^4^CNRS, Institut de Pharmacologie Moléculaire et Cellulaire, Université Côte d'Azur, Valbonne, France

**Keywords:** lipid droplets, molecular dynamics simulations, budding, membrane asymmetry, lipid droplet proteome

## Introduction

Fat storage is an essential mechanism whereby cells store energy that can be later used to perform basal functions when food intake is reduced or insufficient. In cells, fat is deposited in organelles called lipid droplets (LDs). LDs are not mere inert storage pools, but they are active sites of lipid metabolism and remodeling. Furthermore, they are involved in numerous diseases, such as obesity, diabetes, cancer, and viral infection (Welte and Gould, 2017).

Despite this central role in important physiological and pathological processes, the general biology of LDs is poorly understood. This is due to the unique structure of LDs, featuring a core of neutral lipids (NLs), surrounded by a monolayer of phospholipids (PLs). As a consequence of this peculiar composition and organization, the mechanism of LD formation remains largely unclear.

**Figure 1:**
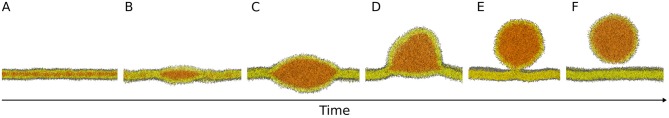
Molecular models of the main steps of lipid droplet biogenesis. Neutral lipids are produced and stored between the two leaflets of the endoplasmic reticulum bilayer **(A)** and they aggregate in lenses **(B)** that grow into nascent lipid droplets **(C)**. Subsequently, lipid droplets bud from the bilayer **(D)** and they can either stay connected to the endoplasmic reticulum **(E)** or detach in the cytosol **(E,F)**. Images are snapshots from molecular dynamics simulations. NL: orange; PL polar heads: gray; PL acyl chains: yellow.

The general consensus is that NLs are produced and stored between the two leaflets of the endoplasmic reticulum (ER) bilayer (Figure 1A); as the concentration of NLs exceeds a certain threshold, they aggregate in lenses (Figure 1B), that grow into nascent LDs (Figure 1C). Subsequently LDs bud from the ER bilayer toward the cytosol (Figure 1D) and, depending on the organism, they can either stay connected to the ER (Figure 1E) or detach in the cytosol (Figures 1E,F) (Wilfling et al., 2014b).

## LD Budding: Evidences and Challenges

The budding step (Figures 1D,E) is crucial for proper LD maturation, and it has important physiological consequences. For example, a budded LD has a higher cytosolic surface that can thus be more efficiently exposed to enzymes, such as lipases, the proteins involved in the catabolism of NLs. Also, enrichment of NLs in the ER is toxic for the cell and formation and budding of LDs might provide an effective mechanism to remove NLs from the ER bilayer (Wilfling et al., 2014b). However, the main forces and molecular actors responsible for the regulation of LD budding are still unknown. Of note, the classical machineries for vesicle budding, such as COPI and COPII, have been ruled out, since, even if COPI can bind to LDs and detach nanodroplets *in vivo* (Thiam et al., 2013), its activity affects protein targeting rather than LD budding (Wilfling et al., 2014a).

On the other hand, regulation of both ER and LD surface tension has been shown to play a crucial role in modulating LD budding (Ben M'barek et al., 2017; Chorlay and Thiam, 2018; Chorlay et al., 2019). To this end, two main mechanisms have been demonstrated to modulate LD budding *in vivo* and *in vitro* by acting on surface tension: (i) protein binding to LDs (Chorlay et al., 2019) and (ii) PL composition (Ben M'barek et al., 2017; Choudhary et al., 2018) and abundance (Chorlay et al., 2019). For example, asymmetry in the PL coverage of the NL core has been shown to favor emergence of LDs, promoting budding toward the side with the higher number of PLs (Chorlay et al., 2019). However, potential mechanisms leading to PL asymmetry between the two ER leaflets, and specifically at sites where nascent LDs are present, are currently not well-understood. Alternatively, asymmetry can also be promoted by protein binding, whereby proteins inserting in the PL monolayer, increase NL coverage and favor budding toward the side where binding occurs (Chorlay et al., 2019). At the same time, PL composition of the ER bilayer can modulate the emergence of LDs from the ER via two distinct mechanisms: PL shape and PL-induced membrane tension. In fact, PLs with intrinsic positive curvature have been shown to favor budded states (Choudhary et al., 2018), as do PLs that are able to reduce ER tension (Ben M'barek et al., 2017).

In parallel, several proteins localized at LDs have also been shown to regulate LD budding. Two such proteins are seipin and Pex30. Pex30 is a membrane shaping protein that can tubulate the ER (Joshi et al., 2016) and that is present only transiently at LDs (Wang et al., 2018). Simultaneous deletion of Pex30 and seipin leads to an impairment in LD budding (Wang et al., 2018). Seipin is a transmembrane ER protein that forms ring-shaped homo-oligomers (Sui et al., 2018; Yan et al., 2018) that can been found stably at ER-LD contact sites (Salo et al., 2016). Cryo-EM structures (Sui et al., 2018; Yan et al., 2018) suggest that the luminal portion of seipin, by covering most of the inner LD monolayer, hinders binding of peripheral proteins toward that side. Therefore, the outer monolayer can be covered by a larger number of proteins, including possibly seipin cytosolic loops, and budding would be favored toward the cytosolic side (Chorlay et al., 2019). Furthermore, electron microscopy images reveal that LD-ER contact sites have a well-defined neck-like topology, and the size of the observed membrane neck is compatible with one ring-shaped seipin oligomer (Salo et al., 2019), suggesting that seipin is crucial to maintain this structure. At the same time, the tertiary structure of the ER domain of the protein is very similar to that of some lipid binding proteins, and it has been shown that the luminal portion can bind phosphatidic acid (PA), suggesting that it could sequester it from the bilayer and possibly present it to metabolic enzymes to form either PLs or diacylglycerols (DAGs) (Yan et al., 2018).

Another family of proteins that is necessary for LD budding is the FIT family (Choudhary et al., 2015). FITs are phosphatases that convert PA into DAG (Hayes et al., 2017; Becuwe et al., 2018), a lipid that not only presents a very low energy barrier for bilayer flip-flop, but that can be also partially stored in the middle of the bilayer, like NLs (Campomanes et al., 2019). Since FITs act only on lipids in the luminal leaflet of the ER, production of DAGs could occur asymmetrically and consequently promote LD asymmetry and budding. At the same time, the high intrinsic curvature of DAG lipids, together with the presence of several transmembrane helices in FIT proteins (Gross et al., 2010), might lead to deformation in the ER cytosolic monolayer generating positive curvature (Thiam and Forêt, 2016). The relevance of deformations in the ER bilayer for LD budding has been proposed also for other proteins that target LDs through a hairpin domain and that, consequently, can impose high positive curvature to the bilayer. An example of this class of proteins is caveolins, also found at LDs (Ostermeyer et al., 2001) and known to deform the membrane at sites of vesicle formation (Parton and Collins, 2016).

## How Can Molecular Dynamics Help Understanding This Process?

From the evidence in the literature, it appears that a combination of protein activity together with changes in membrane properties (such as surface tension, lipid composition, and surface coverage) is key in controlling LD budding. However, several aspects of this process are difficult to address with state-of-the-art experimental methods. Most notably, a detailed characterization of the molecular structures along the budding pathway remains unaddressed and difficult to achieve using current structural biology methods, due to the liquid nature of lipid aggregates, the small size of early-stage nascent LDs (well below optical resolution), and the transient nature of budding intermediates.

Molecular dynamics (MD) simulations are optimally suited to investigate the structural and dynamic properties of liquids, and they are particularly promising for the study of molecular mechanisms underlying LD budding (Soares et al., 2017). Notably, MD simulations have already been successfully applied to interpret and corroborate several experimental findings. For instance, MD simulations clarified how changes in bilayer surface tension alter the concentration of NLs stored in a LD lens (Ben M'barek et al., 2017). Also, MD simulations showed that asymmetry in monolayer coverage (hence asymmetry in surface tension) is able to control budding directionality independently of the lipid spontaneous curvature (Chorlay et al., 2019).

However, several questions remain on the mechanism and the energetics of budding, as well as on the role of different proteins in the process; we foresee that MD simulations will be instrumental in addressing such questions. First of all, MD simulations can be used to explore the structural role of PLs and how the distribution of different lipids influences budding. In particular it will be interesting to understand the role of PLs, such as phosphatidic acid, lysolipids, and DAG, during all the stages of LD growth and budding, since they seem to largely influence budding and protein recruitment (Ben M'barek et al., 2017; Choudhary et al., 2018). Second, MD simulations may help elucidating the energetic requirements associated with various steps of the budding process (depicted in Figure 1). Theoretical studies of LD budding suggest that, in order to achieve LD fission, the NL phase should completely dewed from either the inner or the outer leaflet of the ER, a mechanism that requires external energy, possibly controlled by surface tension (Thiam and Forêt, 2016). We envisage that MD simulations may allow detailed predictions on the energetics of LD budding under different and controlled conditions, therefore clarifying which of the proposed budding stages are spontaneous and which ones require external energy. Third, for those steps requiring external energy input, MD simulations will enable predictions of the molecular mechanisms by which proteins regulate LD budding. For example, how Pex30 and seipin promote concertedly budding is not understood. While it has been shown that seipin imposes a distinct topology to LD-ER contact sites (Salo et al., 2019) it remains unclear if, in order to achieve a fully budded state with a well-defined neck (Figure 1E), the LD lens needs to reach a certain size or if this topology is already stable in the early stages of LD formation (Deslandes et al., 2017).

More generally, open questions remain on the relevance of protein-induced membrane deformations in LD budding as well as on the influence of LD-binding proteins, and MD simulations can greatly contribute to address such questions, particularly as high-resolution structures of the proteins involved become available. Overall, MD simulations can help unveiling which morphologies are more energetically favorable for lipid aggregates with different compositions (e.g., different concentrations of NLs), and which transformations are more likely.

Finally, even though the mechanism of LD formation and budding showed in Figure 1 is generally accepted, whether the final step of the process actually happens *in vivo* remains controversial. Of particular concern, no fission machinery leading to LD detachment from the ER has been identified so far, and it is unclear whether LD detachment could be promoted by membrane physical properties alone. MD simulations should be able to provide an estimate of the energetic cost of breaking the LD-ER neck and to clarify whether the process is driven only by surface tension or if protein activity is necessary to detach LDs from the ER.

## Conclusions

In this Opinion, we illustrate the main unanswered questions regarding LD budding that can be investigated using MD simulations. One of the challenges of simulating such systems is their computational cost, since LDs have diameters of hundreds of nanometers and their growth takes place on time scales of seconds (Salo et al., 2019). The employment of chemical-specific coarse-grained models, such as MARTINI (Marrink et al., 2007; Monticelli et al., 2008) and SDK (Shinoda et al., 2007), has recently allowed simulating some aspects of LD budding using realistic sizes and timescales. However, simulations representing the complexity of LD formation (that involves multiple lipid species and proteins throughout the process) might be beyond the current capabilities and accuracy of available CG models. Equilibrium CG simulations might not be sufficient to explore the key aspects of LD budding, and enhanced-sampling strategies might be required. Thus, even though pioneering simulations have started highlighting important aspects of LD biology (Khandelia et al., 2010; Bacle et al., 2017; Ben M'barek et al., 2017; Vanni, 2017; Pezeshkian et al., 2018; Chorlay et al., 2019; Zoni et al., 2019), we foresee that further developments in molecular modeling techniques will be required to advance our understanding of the mechanisms of LD biogenesis.

## Author Contributions

VZ and SV wrote the article with help from VN, LE, HR, and LM.

### Conflict of Interest

The authors declare that the research was conducted in the absence of any commercial or financial relationships that could be construed as a potential conflict of interest.
